# A Qualitative Scoping Review of Community Firearm Violence in Low-Income Settings

**DOI:** 10.1007/s10900-024-01419-5

**Published:** 2024-10-28

**Authors:** Colleen S. Walsh, Terri N. Sullivan, Wendy Kliewer, Katherine M. Ross

**Affiliations:** 1 Department of Psychology, Vanderbilt University, 230 Appleton Place, Nashville, TN USA; 2 Department of Psychology, Virginia Commonwealth University, P. O. Box 842018, Richmond, VA 23284 USA; 3 Search Institute, 3001 Broadway Street NE #310, Minneapolis, MN 55413 USA

**Keywords:** Firearms, Community violence, Qualitative, Scoping review, Prevention

## Abstract

Firearm violence is a public health crisis in the United States that disproportionately impacts community members in low-income areas who witness and experience violence and violent victimization at elevated rates compared to other socioeconomic groups, often as result of community disinvestment and systemic racism (Smith et al., Soc Sci Med 246:112587, 2020). While quantitative reviews of firearm violence and related factors exist, a review of qualitative methods and findings regarding exposure to firearm violence has not yet been conducted. This scoping review sought to address a gap in the literature by summarizing the findings of *qualitative* studies on community firearm violence in low-income settings in the U.S. EBSCO databases, Criminal Justice Abstracts, National Criminal Justice Reference Service Abstracts, ProQuest, and PsycINFO were searched for studies that described the firearm related experiences of individuals and families in low-income communities. Thirty studies met the criteria for review. Findings were situated within the Centers for Disease Control and Prevention’s (CDC) Social-Ecological Model as a framework for prevention (CDC, The social-ecological model: a framework for prevention, https://www.cdc.gov/violenceprevention/about/social-ecologicalmodel.html, 2018; Dahlberg and Krug, World Report on violence and health, World Health Organization, Geneva, 2002). A critique of the literature, as well as implications and future directions of findings, are discussed. This study may inform future research questions and programs that center the voices of those most impacted by firearm violence.

## Introduction

Firearm injury and death is a prevalent public health problem associated with numerous immediate and long-term negative outcomes [1]. Firearm violence is costly to individuals, families, and communities at an estimated $280 billion annually in immediate (ambulatory) and long-term costs (lost earnings, physical/mental health care) [2–4]. Since 2019, firearms have been the leading modality for three of the top 10 leading causes of death among youth ages 1 to 19 including unintentional injury, homicide, and suicide [5]. Additionally, in the U.S., firearms are the primary modality for three of five leading causes of death for people ages 1 to 44 (i.e., accidental death, homicide, and suicide [2]). These statistics suggest that the impact of firearms on Americans transcends individual demographics, however, significant disparities are seen in rates of firearm death and injury for males (86% of deaths, 87% of injuries in 2020 [2]) and more generally, for youth and young adults of color (in particular Black and American Indian/Alaskan Native groups) who live in low-income communities [2].

*Community firearm violence* encompasses firearm violence experiences such as perpetration, victimization, witnessing, and other exposures (e.g., hearing about firearm violence) within an individual’s community. Members of low-income communities are injured and die as a result of firearm violence in their communities at elevated rates compared to other socioeconomic groups, it is important to consider the specific experiences with firearm violence for these individuals [1]. Additionally, low-income groups face different and additional barriers to services and resources that may act as preventative measures for community firearm violence experiences, making their circumstances unique and the types of supports necessary for curbing rates of community firearm violence to be tailored to community needs [6]. To better understand these experiences, this review focuses on low-income community members’ experiences with community firearm violence.

### Disparities by Race and Community Context

In the past three decades, homicide has been the leading cause of death for Black and African American youth, ages 10 to 24, the majority of which were caused by firearms. In 2020, Black Americans experienced around 40,000 non-fatal firearm injuries a year [7, 8]. Additionally, American Indian/Alaskan Native groups experience the second highest rate of firearm-homicide death [9]. In 2021, Hispanic Americans were 2.33 times more likely to die by firearm homicide than non-Hispanic white Americans [10]. Since 2019, an increase in firearm-homicide has occurred across all racial groups with the exception of Asian/Pacific Islanders [9].

Though all racial groups are represented in low-income communities nationally, people of color including youth, young adults, and families are disproportionately represented as residents in low-income, urban communities that result from systemic racial inequities such as restrictive housing policies (seen in public housing), and community disinvestment (redlining) [11]. American Indian and Alaskan Native groups also face economic marginalization as a result of historic systemic racism (e.g., dispossession of land, economy, wealth, development of reservations, economic disinvestment [12],). In a 4-year study, Gastineau et al. [1] identified the exacerbation of racial and economic disparities during 2020 at the height of the SARS-CoV-2 pandemic period and high rates of firearm violence exposure from 2017 to 2019. Additionally, youth from urban areas (89.3%), southern U.S. census areas (62.7%), and neighborhoods characterized as “very low” on the Child Opportunity Index (higher rates of poverty, inadequate housing, and lower economic opportunity) experienced the highest rate of firearm-related hospital visits. Youth represented in this study were identified by hospital discharge diagnosis International Classification of Diseases (ICD) code (indicating discharge from the hospital following a penetrating injury from a powder-charged weapon), primarily male (78.8%), non-Hispanic Black children (61.7%) [1]. Additionally, exposure to firearm violence mortality (knowing someone who has died from firearm violence) is a social determinant for mental health problems (e.g., psychological distress, depression, suicidal ideation and/or psychotic experiences) for Black and Latine individuals in urban areas [13]. These findings underscore the overlap between individual and community risk for firearm violence involvement and exposure due to systemic socioeconomic inequities including poverty, inadequate housing, and lower economic opportunity [1, 14].

### Contributions of Qualitative Methods to Public Health Research

The high-quality, empirically-based prevention of firearm violence requires an “all hands on deck” methodological approach. Qualitative studies provide both complementary and additional value to quantitative research through a deepened understanding of beliefs, opinions, and feelings about sensitive and often covert behaviors, by the individuals who are most impacted by these issues. Qualitative studies may better define the reasons for firearm violence, as well as the ways in which it is perpetrated [15–17]. Additionally, qualitative methods and findings are important for use in quantitative measure development related to firearm violence prevention.

### Current Study

The voices of youth, families, and communities who are most impacted by firearms can play a meaningful role in the development of interventions and policy solutions to community firearm violence. Insight and context from community voices may be used to confront, address, and heal from the outcomes of racial minoritization and marginalization, as well as inform analytic inquiry [18, 19]. The goal of this scoping review is to summarize the findings of the extant qualitative literature on experiences with firearm violence in low-income communities. Building on the quantitative reviews to date, this study uses a scoping approach to examine firearm violence topics broadly across adolescence and adulthood. Rather than focus specifically on firearm carriage or indirect exposure to firearm violence, this review is intentionally broad to capture salient themes and include the multiple subjective experiences of individuals and families. Synthesis of extant qualitative methods and findings may provide additional context and insight into the intricacies of factors that influence elevated rates of violence in urban, low-income communities of color.

## Method

The studies included in this review were obtained through a scoping review of qualitative studies on attitudes, behaviors, causes, and consequences of community firearm violence in low-income settings.

### Inclusion and Exclusion Criteria

To be included in the sample, studies needed to: (1) include collection and analysis of qualitative data, (2) focus on U.S. samples including youth (10 to 24 years-old) or adults, (3) be written in English, (4) include participants from low-income communities, (5) and report theme(s) and/or subtheme(s) related to firearms. Studies that focused on (1) suicide, (2) veterans and military personnel or service providers (e.g., mental health workers, nurses, first responders), (3) witnessing or experiencing violence online, (4) mass shooting incidents, or (5) intervention/program evaluation were excluded. These topics are also important and warrant independent evaluation to capture the nuance of community member experiences, rather than be included for evaluation in this study.

### Search Strategy

A literature search was conducted on March 27, 2023 in all EBSCO databases, Criminal Justice Abstracts, National Criminal Justice Reference Service Abstracts, ProQuest (which included PubMed based on university subscription), and PsycINFO, using the following search string: AB(“qualitative” OR “mixed method*” OR “mixed-method*” OR “focus group” OR “focus-group*” OR “interview*” OR “ethnographic research” OR “narrative research” OR “thematic analysis” OR “grounded theory” OR “action research” OR “action-research” OR “phenomenological research”) AND AB((firearm* OR gun* OR handgun*) AND (carrying OR carriage OR access OR ownership OR acquisition OR “correlate*” OR “associated*” OR “risk factor*” OR “risk-factor*” OR “protective factor*” OR “promotive factor*” OR “violence exposure” OR “indirect exposure to violence” OR “exposure to violence” OR “witnessing violence” OR “homicide*” OR “injure*” OR “gunshot*” OR “wound*” OR “gunshot*” OR “injury*” OR “shooting” OR prevention)).

**Fig. 1:**
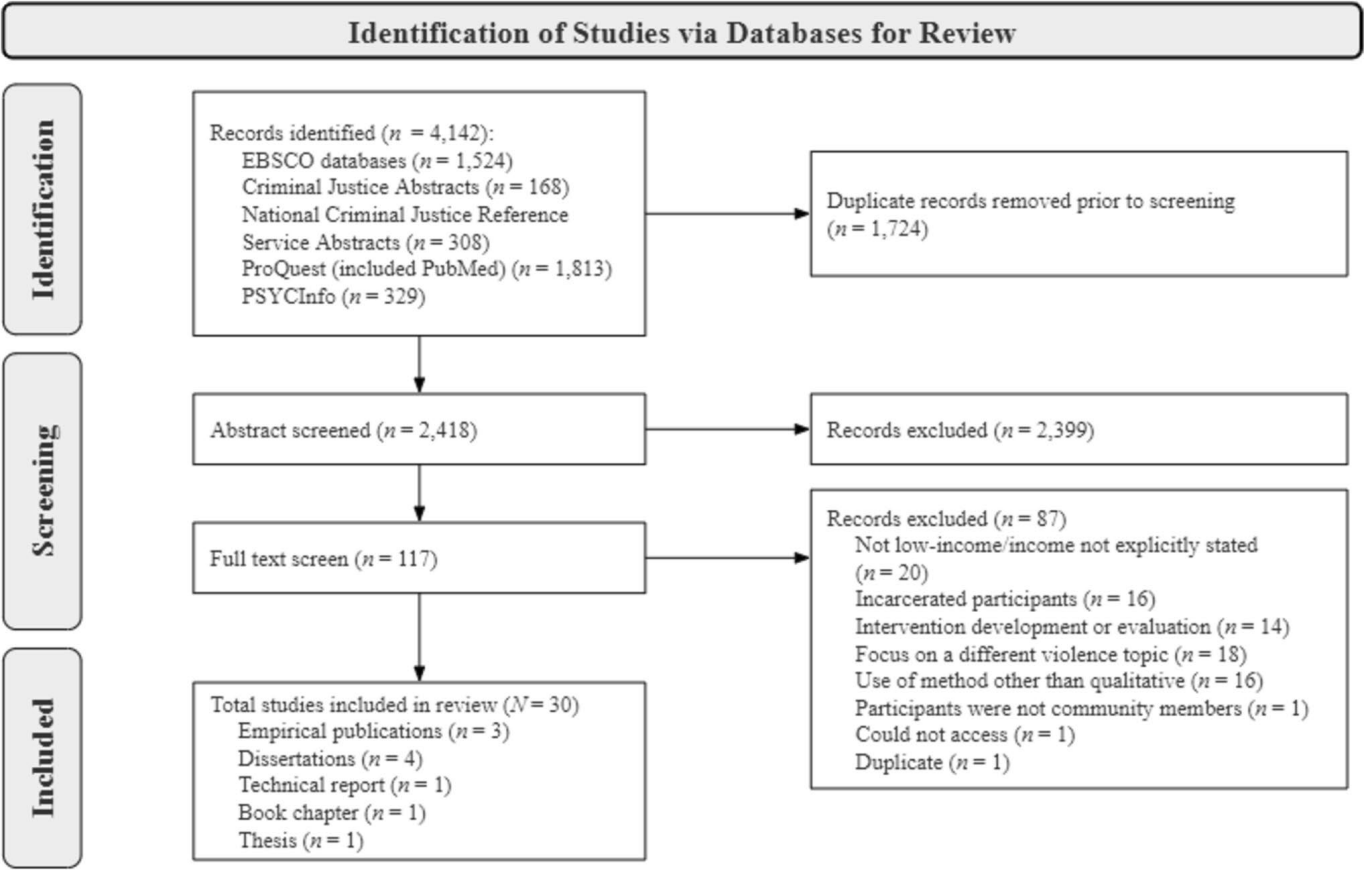
PRISMA flow diagram of search results

The studies included in the review were selected through a multi-stage process [20] that included examining each paper’s (1) title and abstract and then (2) the whole paper. Guided by the methods outlined in [21, 22] a Preferred Reporting Items for Systematic Reviews and Metanalyses (PRISMA) flow diagram for study selection is included to depict the process of identification, screening, eligibility, and included studies (Fig. 1). A codebook for extracting and organizing data was developed by the lead author and was reviewed and approved by the three coauthors. The codebook included sample, setting, qualitative methodology, results, firearm violence experience type, and level of the social ecological model (Table 1).

**Table 1:** Studies included in the literature review (*N* = 30)

Study	Location/setting	Demographics	Qualitative method	Analytic process/theoretic framework	Study aims	Limitations (as reported in study)
[38]	Newark, NJ	*N* = 10 African American males; ages 21–44	Mixed method; interviews	Constant comparative	To explore life post-firearm injury	Phone interviews due to COVID-19; sample size; generalizability
[31]	Southern Chicago, IL	*N* = 5 African American males; ages 22–24	Phenomenological, Semi-structured interviews	Categorization of themes within a pre-existing theoretic framework	To learn the reasons Black male youth and young men kill other young Black males	Only one researcher contributed to the analyses; sample size; researcher bias
[52]	Urban youth leadership program from an urban community-based nonprofit	*N* = 29 youth, ages 14–18; 72% Female; 90% African American, 10% Other race	Focus groups	Thematic	To explore urban teens’ perception of gun violence in their lives, and their ideas for gun violence prevention	Sample size; Racial diversity of participants
[26]	Peer-educator training program sponsored by North Philadelphia Firearms Reduction Initiative, PA	*N* = 30 youth, 66.7% Males; age 13–18	Semi-structured interviews	Not specified	To explore why inner-city youth carry guns, feelings related to gun handling, and how they feel about peers who carry guns	Sample size and age of respondents; Retrospective methodology
[47]	Bronx and Brooklyn, NY	*N* = 108 individuals considered to be high risk for gun violence involvement; *M*_*age*_ = 29.2	Mixed method; In-depth interviews	Grounded Theory	To determine perceptions of and experiences with local underground gun markets	Self-report
[53]	Bronx, Brooklyn, New York City, NY	*N* = 50 young Black men; *M*_*age*_ = 20.9	Interviews	Grounded Theory	To share a nuanced understanding of high-risk men’s’ reluctance to engage with police in shooting incidents	None reported
[34]	Bronx, Brooklyn, NY	*N* = 51 Black men; *M*_*age*_ = 23.1	Interviews	Grounded Theory	To examine high-risk Brooklyn and Bronx residing male’s gun handling and storage behaviors	Generalizability
[39]	Pittsburg, PA	Open-ended responses N = 55, 83.6% Male, 70.9% African American,* M*_*age*_ = 28.5; interviews:* N* = 6, participants were derived from the greater survey	Multiple method: Open-ended responses, interviews	Categorization	To identify important factors for post-firearm injury prevention recovery	Multicollinearity; convenience sample; sample size
[35]	Baltimore, MD	All > 18 years old; 100% Black/African American; demographics not otherwise collected	Mixed method; Focus groups	Grounded Theory	To assess community perspectives on proactive gun law enforcement in Baltimore City	Low participation rates and potential for differences between those who did and did not participate; Incomplete understanding of type of gun victimization
[40]	Level 1 trauma center in a metropolitan hospital, mid-Atlantic	*N* = 16; *M*_*age*_ = 35.5; 81.3% Male; 81.3% Black, 18.8% White	Interviews	Narrative Inquiry	To establish a story of being a victim of gun violence by survivors	Geographic and racial homogeneity of participants; Generalizability
[51]	Micro‐urban Midwestern city	*N* = 11 Black youth; 54.5% Female; *M*_*age*_ = 13.8	Multiple method; Field notes, transcribed group discussions	Constructivist Grounded Theory	To develop a Black, youth informed model of community resilience	Context-specific; Questions regarding youths' experiences with combating violence were not asked; Limited understanding of how community resilience factors operate in the youths' lives; Did not test for gendered differences
[55]	Urban, level 1 trauma center	*N* = 51 gun violence survivors; 84% Male; *M*_*age*_ = 29; 46% African American, 24% non-Hispanic White	Mixed method; Semi-structured, open-ended interviews	Phenomenological Inquiry	To identify individual and community-level factors that affect firearm violence	Generalizability; Single-reporter type; did not include community members < 18yo who do not speak/understand English
[32]	Urban, youth violence prevention program	*N* = 30 youth; 50% Male; *M*_*age*_ = 16.4; 87% African American	Focus groups	Constant Comparative	To understand participants’ experiences with violence	Unable to test for sex, gender, and gendered expression, and romantic relationships related to experiences of violence and Code of the Street
[46]	Four public pediatric clinics, large metropolitan area	*N* = 10 parents; 80% Female; 50% African American, 40% White, 10% Asian	Mixed method; Focus groups	Thematic	To identify characteristics and experiences related to gun ownership an interest in firearm injury prevention counseling for pediatric parents	Adult-report only; Generalizability; Potential falsely negative responses to gun ownership due to sensitive topic
[41]	Level 1 trauma center at Harborview hospital, Seattle, WA	*N* = 60 patients with firearm assault and unintentional injury; representative of this type of patient served by the hospital; 66% Male; *M*_*age*_ = 34; 52% non-Hispanic White, 33% Black	Semi-structured interviews	Categorization of themes within a pre-existing theoretic framework	To explore prior violent exposure, risk, recovery, support services, outcomes and perceptions of firearm violence	Low response and recruitment rate may indicate lack of high-risk patient participation; External barriers to participation for high-risk patients
[45]	Two neighborhoods in Wilmington, Delaware	*N* = 50 street-identified African American women and girls; *M*_*age*_ = 34.3	Mixed method; Semi-structured interviews	Grounded Theory	To examine the impact of gun homicides related-loss on relatives/friends on street-identified Black women	Context-specific; Generalizability
[49]	Urban, Level 1 trauma center	*N* = 29 patients with violent injuries; demographics not otherwise collected	Interviews	Grounded Theory	To identify and characterize strengths and barriers to patient-physician communication in trauma care	Generalizability; Selection bias; No demographics collected; Interviewer bias
[48]	Boston, MA	*N* = 6 African American men; ages including mid- 20 s to mid-30 s	Mixed method; Ethnographic interviews	Ethnographic; Not specified	To develop further insight on gun markets within criminal networks	Self-report; Generalizability
[43]	Denver, CO	*N* = 43 adolescents (10–20 years) and *N* = 42 parent interviews	In-depth interviews	Constant Comparative	To develop narratives of youths’ experience with violence, strategies for coping with violence	Limited types of violence mentioned by participants; Potential for under report of sensitive topics; Limited probing of violent experiences by researchers; Historical timing of interviews (pre Columbine High School shooting)
[33]	Upstate New York	*N* = 9 African American men; 13–19 years old	In-depth interviews	Phenomenological	To explore the symbolic meaning of violence	None reported
[36]	A school in a large Midwestern city	*N* = 20 African American males; ages 14–18	Interviews, case study	Inductive, theoretical	To consider how African American male youth conceptualize safety and subsequent safety behaviors	None reported
[50]	Midwestern university hospital level 1 trauma center	*N* = 10 Black gunshot wound patients from a hospital trauma registry; 90% Male	Mixed method; Interviews	Grounded Theory	To understand post-discharge needs of patients who sustain violent injury and their families	Small, homogeneous sample
[54]	New York City, NY	Interviews *N* = 69 NYC Housing Authority residents; 60% Female; *M*_*age*_ = 39; 73% Black, 14% Latinx Focus groups* N* = 38; 55% Female; *M*_*age*_ = 36; 70% Black, 15% Latinx	Multiple method; In-depth interviews, focus groups	Thematic	To understand perceptions of violence following the COVID-19 outbreak	Historical timing of interviews (vaccine requirements, mandates); Only recruited to English speakers living in NYC Housing Authority sites
[42]	Urban, municipal hospital in Boston	*N* = 18 African American men who had been shot or stabbed; Ages 18–25	Open-ended, semi-structured interviews	Not specified	To explore experiences with violent injury among individuals with gunshot, stab wounds	Generalizability; Not randomized or consecutively selected; Context-specific
[27]	Oakland, CA	*N* = 11 African American males	Semi-structured interviews	Grounded Theory	To explore perspectives and experiences that influenced their gun violence behaviors	Self-report; recruitment and retention
[44]	Southcentral Los Angeles, CA	*N* = 419 African American mothers of newborns; *M*_*age*_ = 24.8	Open ended survey questions	Classification	To determine what mothers fear for their newborn’s future	Participants may have shared more detailed concerns with more ample opportunity for reflection
[37]	5 elementary schools, medium sized city, Pacific Northwest	*N* = 20–25 children engaged in D.A.R.E at each school; Ages 8–12; demographics not otherwise collected	Multiple method; Discussion groups	Content analyses	Sought to examine low-income household practices of firearm possession and storage	None reported
[28]	Western Baltimore, MD	*N* = 18 African American males	Descriptive case study, interviews	Categorization of themes within a pre-existing theoretical framework	to explore perspectives and experiences that influenced gun violence behaviors	Researcher bias; sample size and diversity; generalizability
[29]	New York City, NY	*N* = 377 active violent male offenders; *M*_*age*_ = 19.3; 44.8% African American, 41.6% Puerto Rican, 13.6% Other Caribbean or mixed ethnicity	In-depth interviews	General inductive and deductive methods	To understand violence as a social interaction and incorporate the symbolic interactionists perspective within adolescent development in order to understand the meaning and function violence may have for youth	Convenience sample; breakdown in sampling/screening procedures, developmental nature of measurement protocols, interviewer-research bias, skill/ability of interviewers over time
[30]	New York City, NY	*N* = 416 active violent male offenders; *M*_*age*_ = 19.5; 48.2% African American, 39.7% Puerto Rican, 12.1% Other Caribbean or mixed ethnicity	Interviews	Thematic	To examine the role of peers in patterns of gun-related behavior	Self-report; Generalizability; Cross-sectional, no follow up

## Results

Of the 30 studies that met inclusion criteria for review, four (13.3%) were published between 1962 and 1998, seven (23.3%) were published between 2000 and 2017, and 19 (63.3%) were published in 2018 or later.

### Characteristics of the Reviewed Studies

One third (*n* = 10, 33.3%) of the reviewed studies included youth in their sample. The remaining studies focused on adults (*n* = 20, 66.7%), a small portion of which sampled parents specifically (*n* = 3, 9.4%). Approximately 40% of studies sampled exclusively males (*n* = 13, 43.3%) and only two studies sampled only females (6.7%). Five studies did not report sex demographics (16.7%), and the remaining studies sampled males and females (*n* = 11, 36.7%) wherein 20% (*n* = 6) were majority male samples. Most studies sampled African Americans, specifically 26.7% (*n* = 8) reported African Americans as their largest percentage of study participants and 50% (*n* = 15) included exclusively African American participants. One study (3.3%) included a predominantly white sample. Five studies (16.7%) did not include race or ethnicity demographics. Finally, all studies included in this review focused on low-income samples (i.e., samples specifically referred to as low-income, reported as at or below the federal poverty level or unemployed or illegally employed) or settings (i.e., data collection in public housing neighborhoods). Relevant data is charted in Table 1.

Study locations included hospitals or clinics (*n* = 6, 20%) and education settings or programs (*n* = 4, 13.3%), as well as one nonprofit (3.3%). The remaining studies’ data collection occurred in urban community settings (*n* = 19, 63.3%). Most studies specified the city of data collection (*n* = 20, 66.7%). New York City, New York (*n* = 5, 16.7%), Baltimore, Maryland (*n* = 2, 6.7%), and Boston, Massachusetts (*n* = 2, 6.7%) were most frequently reported (see Table 1).

The final sample of reviewed studies consisted of 18 (60%) qualitative studies, eight (26.7%) mixed method studies, and four (13.3%) multiple method studies. Multiple method studies are categorized separately from the mixed method studies because: 1) the authors did not identify their study as mixed method and 2) the studies did not include a “convergence” component to their analysis, which is traditionally considered critical of mixed method research [23]. Data for the included studies were primarily collected through interviews (*n* = 23, 76.7%), though five studies (16.7%) used focus groups, and two (6.7%) used multiple data collection methods. The latter two studies included either an interview or a focus group in addition to another qualitative method (e.g., field notes, journal entries; see Table 1).

Several theoretical and analytic approaches were used in data analyses across the included studies. Grounded theory or a modified version of grounded theory was most frequently used (*n* = 9, 230%). The next most frequently used approaches included, thematic analysis (*n* = 4, 13.3%), categorization (*n* = 4, 13.3%), constant comparative analysis (*n* = 3, 10%), phenomenological inquiry (*n* = 2, 6.7%), narrative inquiry (*n* = 1, 3.3%), classification (*n* = 1, 3.3%), and content analyses (*n* = 1, 3.3%). Five studies (16.7%) described their analytic process to some extent but did not reference or report a specific theoretic or analytic approach. Relevant data is charted in Table 1.

### Summary of Findings

The CDC’s Social-Ecological Model was used to frame the results of this scoping review [24, 25]. In this conceptualization, the Social-Ecological Model considers the interplay of individual-, relationship-, community-, and societal-level factors associated with community firearm violence and related behavior. This framework posits that each level influences the others and that in order to develop contextually relevant community firearm violence prevention practices, scientists must simultaneously target factors across the levels of the model. Eighteen studies examined factors at the individual level, 10 at the relationship level, nine at the community level, and eight at the societal level. Some studies include themes that covered factors at more than one level of the Social-Ecological Model. A summary of individual study findings are described in Table 2.

**Table 2:** Main findings from qualitative research on firearms in low-income settings (*N* = 30)

Study	Firearm Topic	Primary Themes or Findings
[38]	Survivorship	Themes summarize the ways men’s live were impacted post-firearm injury, development and adjustment to long-term disability and the physical injuries, changes in how masculinity is expressed (gender norms and roles, social identities) following injury, and impact of firearm violence and injury on mental and emotional health
[31]	Motivation; Risk & resilience; Community-specific norms	Themes of motivation and antecedents for murder included a culture of violence, traumatic experiences, single parent households/lack of support, insecurity, fear, and lack of confidence, supporting the family in lieu of a father figure, modes of masculinity and respect, persistence, resilience, and humor, poor education experiences, and conflict avoidance
[52]	Police, Solutions to firearm violence	Two primary themes: gun violence was linked to racism and poor relations with the police. Youth reported a need for improved relations with police and the importance of personal relationships with adults as a means for decreasing gun violence
[26]	Motivation, Behavior; Peers; Police	Frequently reported reasons for gun carrying included protection during drug dealing, protection from disrespect (including bullying and aggression). Emotional responses to gun carrying primarily reflected experiences of fear and excitement. Inconsistent perception of peer attitudes towards gun carrying. Specified a specific form of gunplay, called “flossing” (playing and/or spinning a gun, mimicking scenes, shooting bullets in the air)
[47]	Gun markets	Two primary themes: firearms Acquired and Immediate Acquisition Pathways & Flow of Guns into the NYC
[53]	Police	Determined that participants are reluctant to cooperate in a shooting investigation due to mistrust of police as a result of under- and over-policing; feelings of legal cynicism, “beefs, gun violence; preference towards “self-help”, or retaliatory violence, rather than cooperation; Concerns with consequences for cooperation
[34]	Behavior, Risk & resilience	Themes include: extreme risk of gun violence, lack of formal gun training, unsafe firearm storage, firearm sharing
[39]	Survivorship; Healthcare and post-injury discharge needs; Health insurance; Police	Primary findings from open ended questions included frequencies that indicated that the physical and psychological healing was the most difficult aspect of recovery; the most positive aspect of recovery included no positive aspects or living each day to the fullest; Participants reported that their relationships with others either did not change or they do not trust others; Participants shared that the greatest sources of strength in their recovery came from immediate family, family and friends, themselves, God, or they did not know. Participants reported their immediate emotional and behavioral responses to being shot, institutional responses (medical care, psychological counseling), police and legal responses, hospital billing and insurance, social support (changes in relationships, family and friends), and searching for meaning (“why me?”, positive change in outlook)
[35]	Feelings of safety; Police	One quarter of participants were victims of a crime with a gun, the majority reported feeling safe in their neighborhood, but less than half believed the police would respond quickly to gunfire. Participants expressed that individual- and institutional-factors influenced their safety. Some participants reported being harassed or treated poorly by police, and reported distrust for police due to ineffective efforts to reduce gun violence in the community
[40]	Survivorship	Pervasiveness of everyday violence, feeling left out, or left behind by society, reactive violence fueled by poverty and limited employment/education opportunities, mental health impacts after gun violence
[51]	Restrictions and access to Positive Youth	Development of “Power through Black Community and Unity (PTBCU). PTBCU is comprised of distinct components: collective care, “seeing beyond the bad”, and facilitation of supportive teen-centered spaces
[55]	Solutions to firearm violence	Factors that contribute to community gun violence included: community problems, happenstance “being in the wrong place at the wrong time”, lack of conflict management and mediation skills.Proposed solutions included: improving employment and education opportunities, community outreach, conflict management and mediation, and increased police presence
[32]	Motivation	Themes of youth experiences specific to peer decision-making related to violence: youths’ want to be respected, youth want to be respected as a means to feeling safe, youth identify guns as a potential risk to their safety, and youth expressed conflict and frustration in balancing the need for respect and personal safety in an unsafe, limited opportunity setting
[46]	Ownership	20% of parent participants had a firearm in their home; 25% had a family member who had been shot; 82% reported being interested in safe gun storage information; 47% would adhere and 37% would consider a pediatric care provider’s advice for safe storage; Of gun owners, 19% would follow provider advice, and 55% would consider it. 6% reported they would ignore or be offended by provider advice about safe storage
[41]	Risk & resilience; Healthcare and post-injury discharge needs	Themes included: half of the sample endorsed prior violent exposure; General societal violence, unsafe communities, and firearm practices facilitated feelings of risk; Key components to recovery included family and social support, mental health services, financial support services; Consequences of violence included psychological symptoms, changes in relationships, and developing a new purpose or mission in life; Overall negative views of firearms, and endorsement of restricted access and firearm safety; connected lack of community opportunities to firearm violence; general disappointment in the legal system
[45]	Loss of a loved one	Younger girls were more likely to report having experienced multiple losses of loved ones to firearm-homicide. Participants reported the comfort in their social environment and general feelings of safety changed following the loss of their loved one in that neighborhood safety and desirability of the neighborhood decreased. Participants reported knowing that Black males were at highest risk of firearm-homicide, but that their fear of victimization had increased since the loss of their loved one, and subsequently felt the need to isolate and withdraw from public spaces
[49]	Healthcare and post-injury discharge needs	Expressed three primary themes including, challenges in communication between patients and physicians, emotional responses to gunshot wound trauma, and strengths in patient-physician communication
[48]	Gun markets	Individuals involved in gangs or drug sales pay inflated prices for trafficked handguns and higher-caliber semiautomatic weapons
[43]	Risk & resilience; Families	High and medium risk youth reported higher rates of turning to friends as a means for coping with violence than low risk youth. This finding is rationalized that youth who are high or medium risk may believe their peers (older youth, gang members) are knowledgeable about handling violence. Connected increased firearm access or carrying, or violence was related to access to positive youth development and other opportunities in a given neighborhood
[33]	Motivation	Symbolic meaning of violence included violence for establishing respect, intimidation and self-defense or protection
[36]	Feelings of safety; Risk & resilience; Peers; Adult relationships	Cognitive geocoding revealed that youth strategically map out safe and unsafe locations in their community as a means of avoiding violence victimization. Decisions about safe and unsafe locations were based on (1) knowledge of the neighborhood, (2) positive and negative peer and adult relationships, (3) the youths’ future orientation and goals
[50]	Healthcare and post-injury discharge needs	Seven primary themes including: feelings of stigmatization by hospital employees, patient-provider communication, concern with discharge timing (too soon), challenges obtaining medicines, challenges with police, transportation for follow-up care, and worry for returning to their community. Participants expressed the need for mental health care for themselves and their family, assistance with paperwork, and more follow-up support after discharge
[54]	COVID-19 impact on violence; Solutions to firearm violence	Two primary themes related to perceptions of violence following the COVID-19 outbreak: social and health impact on perceptions of violence and safety, and community recommendations for reduction of violence
[42]	Survivorship	Established the concept of “being a sucker”—a person who does not retaliate after being disrespected or injured. These people are subsequently viewed as weak and are targets for future violence victimization
[27]	Motivation; Behavior; Police; Solutions to firearm violence	Causes of gun violence included job markets, drug market participation, unstructured time, single parent households, anger, hopelessness, realization, and illegitimacy of policing. Gun acquisition, access was facilitated by male peers and male family members. Men reported that guns facilitated feelings of power, control, and protection. Situational factors included rational choice, physical location, collective efficacy and informal social control. Prevention strategies and programs for gun violence included employment, educational opportunities, resources, African American Fathers and Role models, restorative justice circles, CPTED, community-police relationships
[44]	Families	39% of mothers reported fears for their newborns: gangs and/or violence, health concerns, drugs and alcohol, growing up in their particular community, and society/the world in general. Fear of gangs and/or violence was 50% from mothers of male newborns, compared to 28% of mothers of female newborns
[37]	Feelings of safety, Risk & resilience	Participants reported on risky firearm behavior in low-income homes and communities such as unsecured firearms, gunfire, finding bullets and cartridge casings. Participants expressed that firearms make them feel safe – though also reported that school feels safe because it has a no gun policy
[28]	Survivorship; Risk & resilience; Peers; Family	Participants discussed the bidirectionality of psychological and behavioral conditions with gun violence experiences across their relationships
[29]	Motivation; Behavior; Risk & resilience; Peers	Social Interaction themes: weapon details, role of the respondent and opponent, relationship between the respondent and opponent, the role of third-party individual, reasons for the violence, location/context, drug use, and the police
[30]	Motivation, Risk & resilience; Peers	Self-protection and safety were the most frequently stated reason for peer gun carriage and possession. Guns and facilitated risk for deadly conflict, and peers were involved as co-offenders in gun related incidents reported

#### Individual Level Firearm Factors

Individual level factors related to firearms include a variety of personal processes and experiences related to the increased or decreased likelihood of firearm violence victimization or perpetration. The majority (*n* = 18) of the reviewed studies explored individual level factors, which are detailed by subheading.

*Motivation* Commonly reported motivation for involvement in firearm violence included protection or safety related to drug sales or purchase [26] and interpersonal conflict and disrespect [26–30]. Studies of youth motives for firearm use included pursuit of respect from others and personal safety, including self-defense and intimidation [27–29, 31–33].

*Firearm Behavior* Several studies sought to characterize firearm behaviors such as firearm carriage, handling, and storage [26, 27, 29, 34]. In these studies, emotional responses to carrying a gun were identified including excitement, power, safety, and fear of being caught [26, 27]. One study reported that the majority of their participants, despite having been identified as “high-risk gun carriers”, never received formal instruction for gun carrying or use [34]. This study also reported that participants frequently stored guns in unsecure, easily accessed areas.

*Feelings of Safety* Three studies found that youth reported feeling safer when guns were present [35–37], though in one case, middle school students from high-violence areas reported feeling an increased sense of safety at school where guns were not permitted [37]. Patton [36] used a novel analytic technique called cognitive geocoding, which used youth participant response to strategically map locations within the youths’ communities that they identified as safe and unsafe. Safety and lack of safety in a given area was determined based on the youths’ calibration of whether or not they were likely to be violently victimized in that area. Three primary appraisals were reported by youth as the criteria by which they determined a locations safety status, (1) knowledge of the neighborhood (i.e., places where the youth or someone else had been victimized), (2) knowledge of peers and adults in each location who might be able to help or were inclined to harm, and (3) the development of personal hardiness and focus on long-term goals (discussed further in risk and resilience) [36].

*Survivorship* Six studies centered on gun violence *survivorship* such as prior experience with community violence, beliefs about why violence occurs, behavior expectations post-injury, adjustment to life post-injury, and considerations for life after hospital discharge [28, 38–42]. Four studies conducted data analyses in a healthcare or hospital setting [39–42], one used phone interviews due to COVID-19 safety precautions [38], all with exclusively, or primarily African American male participants. A common theme expressed by participants across studies were the prevailing rates of everyday violence, wherein violence was characterized as reactive or retaliatory [28, 38–42]. One study focused on the perceived need to not be a “sucker” or someone who does not retaliate with violence after being violently injured [42]. Participants also described the high rates of violence in their communities as a result of poverty and lack of opportunity [28, 38, 40]. In some cases, participants discussed feelings of abandonment by institutions and society (e.g., hospital, legal systems) and fear of returning to the community after discharge from the hospital [40, 41]. Participants from four studies discussed emerging personal outcomes stemming from injury or violence exposure including new physical challenges (disability, long-term injury [38, 39],) and psychological effects including PTSD anxiety and hopelessness [28, 38–41], changes in relationships (i.e., loss of trust), and a new sense of purpose or mission in life [38–41].

*Risk and Resilience* A number of studies explored and identified aspects of risk and resilience related to firearm violence [28–31, 34, 36, 37, 41, 43]. In the majority of these studies, participants reported themes of exposure to violence through witnessing or hearing about violence, or being victimized [28, 31, 34, 37, 41, 43]. Other elements of risk for firearm violence involvement included aspects of the individual’s community (e.g., high rates of violence,[28, 29, 31, 36, 41],firearm sharing; [34],finding bullets or cartridges casings; [37]) or personal practices (informal firearm training, unsafe storage [34],). Irwin organized youth and parent assessments to categorize youth as high, medium, and low risk, and from these categories, youth in high and medium risk categories reported higher rates of turning to friends as a means for coping with violence as compared to youth in the low-risk category. Youth who are at high or medium risk may believe their peers (older youth, gang members) are street-savvy or knowledgeable about handling violence [43]. In that same study, Irwin [43] reported that parents across risk groups were often unaware of their youth’s experience with or exposure to violence. Participants from Barnes [31] reported a culture of violence in their communities, lack of educational opportunities, limited family support (i.e., single parent household, having adult roles or expectations as a child), and personal characteristics (insecurity, fear and low self-confidence) were risk factors for engagement in community firearm violence. This study also reported that in lieu of these challenges, participants also reported aspects of their own resilience including persistence and humor, and conflict avoidance. Another study found that the development of personal hardiness (i.e., commitment to control over emotions despite challenges or threats) and maintaining a positive sense of self by focusing on personal goals (i.e., graduating from high school) were aspects participants identified as ways they coped with living in their high-violence communities. Several of these studies discussed adaptive coping behaviors by participants including conflict avoidance [31], physical location avoidance (i.e., high-crime areas [36, 43]), and radical acceptance of poor community conditions [43].

#### Relationship Level Firearm Factors

Relationship level factors related to firearms include a variety of interpersonal processes and close relationships with peers and family associated with the increased or decreased likelihood of firearm violence victimization or perpetration. While some of the findings from these studies can be classified as an individual’s experience with firearms, they have been categorized as relationship level factors to reflect the reporter’s relationship to someone who has or may experience gun violence, (*n* = 10).

*Peers and Families* Relationships with peers influenced youths' firearm behavior. The study by Black and Hausman [26] focused on a community sample of youth and interviews revealed that youth believed that their peers respected people who carry guns and felt protected when guns were around. However, youth from the same sample reported that they themselves were indifferent, or fearful for peers who carried guns, illustrating a disparate understanding of peer-perception, though youth who reported they were non-carriers were more likely to endorse fear and carriers were more likely to report indifference or respect. Hansen et al. [32] expressed similar themes pertaining to peer influence on firearm behavior and added that youth balanced the need to preserve their own safety with the pursuit of respect from peers that they believed came from carrying a gun or engaging in violence. White [28] similarly reported that individuals who had experiences with gun violence reported having been influenced by peers, and family members, who used firearms and modeled violent behavior (resolving conflict with a firearm). Similarly, Wilkinson and Fagan [29] discussed the influence of peers as “bystanders” in incidents of violence. In these cases, youth reported that bystanders had been present and encouraged the youth to engage in violence against another person, and rarely had discouraged or sought to break up violent incidents. Wilkinson et al. [30] found youth who identified as active violent offenders reported high rates of peer-gun carriage. The most endorsed reasons for peer-gun carriage were for protection, drug trade, to avoid beef (e.g., conflict), and because it is cool. Yet, only 13.7% of youth in this sample said that all of their peers carried guns, indicating that the vast majority of youth in this sample associate with peers who do not carry guns [30].

*Experiences with the loss of a loved one* Two studies reported on the fears and the way parents cope with fear that they have for their children’s safety within their community neighborhood setting. Schuster et al. [44] found 40% of mothers in their sample (all African American) reported fear of gangs (including drive-by shootings, getting shot in crossfire), violence, or both, though 50% of mothers of newborn boys reported these fears, compared to 28% reported by mothers of newborn girls. Patton [36] shared the narrative of a youth who reported their father had advised them on how to manage unsafe locations where it was likely they could be victimized. The youth reported that their father had taken him around the neighborhood and introduced him to friends and former gang-related associates as a way to give the youth a network of people who would look out for him and who the youth could contact if he needed help or protection.

One study considered the perspective of third-party individuals, namely the cumulative effect of the loss of a loved one to firearm-homicide on street-identified Black women and girls [45]. “Street-identified” in this case, was defined as those in close proximity to “street-life”, or those who honor the expectations of “street code.” Younger girls (16–24) were more likely to report having experienced multiple losses of loved ones to firearm-homicide, most of whom were Black men. Participants reported their comfort safety in their social environment changed following the loss of their loved one, such that their perception of neighborhood safety and the desirability of the neighborhood decreased.

*Ownership* Finally, one study [46] considered parents’ characteristics and experiences with firearms to determine their attitudes towards in-home firearm safety counseling. The sample represented parents classified as low-income who bring their children into urban pediatric clinics. Primary thematic findings indicated that all parents (80% female) in the sample worried about protecting their home and family. Not all parents reported keeping a gun in the home as an effective means of safety, and all parents reported alternative methods of home safety (mace, alarms, dogs) as inopportune, as they are expensive or ineffective. The majority of parents reported interest in firearm injury prevention counseling and that they would like to receive more information about safe firearm storage. Nearly half of parents reported they would follow physician suggestions to not keep a gun in the home, though 37% reported they would consider the recommendation only 19% of gun owners endorsed considering the recommendation.

#### Community Level Firearm Factors

Community level factors related to firearms include a variety of settings and characteristics of settings that are associated with frequency of violence in the community and experienced by community members. Nine of the reviewed studies discussed community level factors related to firearms.

*Gun Markets* Two studies discussed community-level issues of firearm *gun markets* [47, 48]. Both studies centered primarily on the experiences of individuals who had experiences in drug- or gang- related behavior and were focused uniquely on the trade and sale of firearms in specific communities. In a study of experiences with illegal gun markets in the Bronx, New York City, participants reported reasons for illegal gun purchase (e.g., self-defense, social status, crime), types of illegal firearms purchased (e.g., handguns, semi-automatic pistols), and reported that in many cases illegal firearms were not purchased with money, but rather were borrowed, and that gang members have established supply lines [47]. Braga et al. [47] also discussed social connections as a means of access for gang and non-gang members, and that most participants perceived that illegal guns were streamed into their community from states with less restrictive gun laws. Additionally, Hureau and Braga [48] found that, in a sample of young Black men in Boston, participants shared that firearms possessed by gang members had come from outside of the state. This study also reported that gang members and drug dealers are exploited by firearm traffickers, who enforce inflated prices for firearm purchases.

*Healthcare and Post-injury Discharge Needs* Four studies explored the barriers and supports to *healthcare and post-discharge needs* of adults with violent injuries [39, 41, 49, 50]. All samples included or were solely comprised of patients with gunshot wounds (GSW). Challenges faced by participants within the healthcare setting post-injury included patient-provider communication (e.g., patients feeling stigmatized, ignored, or misunderstood, physicians providing inadequate information [49, 50]), discomfort with police presence in the healthcare setting and inadequacy of transportation for attending post-discharge appointments [39, 50], and patient and physician emotional responses to trauma that hindered care and communication (e.g., feeling like they are living in “survival mode” or “war-like” conditions, posttraumatic responses [49]. One study discussed the financial strain and pressure they experienced as a result of “enormous” hospital bills and insurance claims following discharge from the hospital [39]. Additionally, Patton et al. [50] found patients in their sample, all Black adults with GSWs, faced challenges with obtaining medications and transportation to appointments following discharge, and were concerned about being discharged too soon and worried about returning to the community. Huang et al. [49] also reported the need for connection between patients and physicians following violent injury including shared social and cultural backgrounds between patients and physicians, open-mindedness and empathy, listening, understanding and managing stressors, and receiving quality care. Two studies reported that participants believed important aspects of their recovery post-injury included family/social support, mental health care, and financial support services [39, 41].

*Restrictions and Access to Opportunities* Parents in low-income communities reported that affluent communities had more recreational opportunities for youth than exist in their communities [43]. Grant et al. [51] used a community sample of youth to inform and develop a youth-centered model of community resilience, “Power through Black Community and Unity”, wherein resilience for communities impacted by gun violence included collectivist practices of mutual care and support and the facilitation of supportive teen spaces.

*Community norms* One study detailed community-specific norms related to firearms [31]. In Barnes [31], male participants, all of which were on probation, discussed a “culture of violence” and trauma wherein “every facet” of their life had included an aspect of firearm violence (i.e., witnessing violence and experiencing firearm violence in and out of the home and neighborhood). Participants from this study discussed firearm violence as an aspect of socialization with peers and within family structures, as well as an aspect of survival.

#### Societal Level Firearm Factors

Societal level factors related to firearms include a variety of broad macro factors that facilitate a climate in which violence is supported or discouraged. These factors may include social norms, as well as disparate population-level health, economics, education, and politics. Eight of the included studies explored or identified societal level factors.

*Police* Several of the studies in this section discussed *police*. Each of these studies included individuals who either lived in high-violence communities [27, 35, 52, 53], or had sustained a gun violence injury [39]. These studies shared common themes, including participant report that negative interactions with law enforcement shaped beliefs about the illegitimacy of police authority and the law in general, ambivalence about police presence in their communities, and increased their legal cynicism and decreased perceptions of fairness [27, 35, 39, 52, 53]. Two studies specified that individuals reported mistrust of police as a result of over and under policing, and that feelings of mistrust positioned participants to be non-cooperative with police when gun violence occurred in their community [39, 53]. Of the studies focused on police factors, one focused on a youth sample [52]. Youth reported that they do not have positive relationships with police communities, in part as a result of unfair treatment of people in their neighborhoods by police, police use of force, and institutional racism [52]. Crifasi et al. [35] further found that racism played a part in the communities’ perception of police as it pertained to firearm violence. More specifically, participants (100% Black/African American) reported being treated poorly by police as a result of being Black, or because of the power police believe they have over people in their community.

*COVID-19 Impact on Firearm Violence* One study provided insight into New York City residents’ perceptions of *violence following the start of the Coronavirus (COVID-19) pandemic* [54]. Study participants reported an increase in gun violence in their communities and feeling that safety had been compromised because of the pandemic. Participants reported that because of stay-at-home orders, youth had fewer structured activities to partake in and were outside of their homes, causing an increase in stress and worry for safety by parents. Inversely, some participants perceived that there were more incidents of domestic violence in the community due to stay-at-home orders, which increased the opportunity for in-home conflict. Patton et al. [54] also reported that participants felt that the Black Lives Matter protests following the murder of George Floyd by a policeman, which occurred in tandem with COVID-19, facilitated their increased displeasure with police and decreased feelings of safety.

*Solutions to Firearm Violence* In some cases, participants offered *recommendations* to manage or amend societal factors that affect firearm violence in low-income communities. Participants reported that gang and drug activity where firearm violence is likely, would be less appealing if job and educational opportunities were more available [27, 55]. Halimeh et al. [55] also reported that participants believed issues of firearm violence should be “dealt with” by the impacted communities in partnership with the broader community and hospital trauma centers. Suggestions related to issues with police included improved accountability and additional training in how to work with the communities they serve [35]. Youth offered that police should seek to develop positive, long-term relationships between themselves and young people in the community, and that there should be increased opportunities for adult mentorship from adults who looked like them (youth) in general [52]. Participants reported the need for African American mentorship programs and role models, as well as non-violent conflict resolution methods [27]. Additionally, Patton et al. [54] prompted participants recommendations for how to increase ethical policing and decrease gun violence. Study participants offered direct and indirect solutions including training for police that addresses racial bias and increases the capacity to support individuals in mental health crisis, redistribution of police funding to communities, increased building security, community resident patrols and interventions for those in distress increased mental health programs, improved housing stability and living conditions, and youth programming [54].

## Discussion

This scoping review summarizes the breadth of qualitative literature on community firearm violence in low-income settings in the U.S. Findings provide complimentary insight to quantitative reviews and findings and may elucidate the multiple subjective firearm related experiences had by individuals who are most impacted by community firearm violence and can share humanity and insight to this complex public health problem. Community firearm violence intervention and prevention efforts have been on the rise in the U.S., as seen in the increase of qualitative firearm related studies published since 2018 (50% of the reviewed sample). This uptick in research is timely and important. To inform future studies of community firearm violence in low-income contexts we share several considerations:

### Consideration 1: Center those who are Most Impacted by Community Firearm Violence

Most studies included in this review focused on Black and male individuals which is important because of the overrepresentation of Black Americans in low-income communities, and their high rates of firearm victimization. A continued understanding the unique experiences of these individuals is needed. However, as established by population health data [5, 9] there are several other demographic groups that are highly impacted by firearm violence in low-income contexts but are not represented in the reviewed studies. For instance, Latinx, Native Alaskans, and immigrant groups are also disproportionately represented in low-income communities and experience high rates of firearm violence. Additionally, it may be important to expand our understanding of the experiences of women and girls (inclusive of gender identity) with community firearm violence exposure, as only two studies used exclusively female samples (e.g., street-identified Black women and girls, and mothers of newborns; [44, 45]). Understanding the perspectives and experiences of these groups can provide more well-rounded insight into the conditions in which firearm violence occurs and be used to garner potential prevention efforts.

Additionally, a qualitative, developmental perspective may provide further insight into the patterns and changes in firearm violence experiences had by young people in low-income contexts. Only one study reported differential experience based on age, specifying that younger girls (16–24) (relative to older women) were more likely to report having experienced multiple losses of loved ones to firearm-homicide [45]. By examining specific developmental groups (e.g., adolescents, young adults) or life stage (e.g., parenthood) overtime we may begin to understand specific pattens or changes in motivations or behavior over time. For instance, it may be important to understand how and why youths’ motivation for firearm carry changes as they transition out of adolescence and into young adulthood. Longitudinal developmental qualitative explorations may lend themselves to the adoption of a strength-focused lens. Which may aid in specifying specific aspects of young peoples on individual- (e.g., identity, self-esteem), relationship- (e.g., positive peer networks), community- (e.g., community cohesion), or societal- (e.g., funding for positive youth development opportunities) factors life that act as buffers or prevention of firearm violence experiences.

### Consideration 2: Explore and Specify Antecedents of Community Firearm Violence and Related Behavior

The bulk of the reviewed studies focused on communities’ experiences with the outcomes of firearm violence. To shift to a prevention lens, it is additionally important to consider the precursors to violence. For instance, reasons for acquiring, owning or carrying a firearm have been modestly documented in quantitative and qualitative literature, though mostly descriptively. It may benefit prevention efforts to understand how individuals in low-income communities appraise threat and how that relates to their perception of need to own or carry a firearm. Additionally, understanding reasons individuals in these communities chose to *not* acquire or own a firearm is perhaps of further importance.

No studies in this review spoke to experiences with social media as a component of community violence. Some scholars have proposed that social media acts as a vector for in-person community violence by providing an additional, non-time constrained platform for starting or exacerbating violence through images, videos, comments and messages that may lead to firearm violence outcomes [56], Author reference). Social media text analyses (method not included in this review) have been conducted, but interview and focus group style data collection may provide additional insight into these experiences within low-income contexts.

### Consideration 3: Consider the Processes of Socialization Related to Firearm Violence and Safety

A third of studies in this review considered relationships between individuals (e.g., [28], or their experiences with community firearm violence in their social role as a peer or caregiver (e.g., [44]), and in one case a study focused on community norms [31]. That said, both the qualitative literature to date has not substantively considered socialization behavior of families and their youth as it pertains to firearm violence and safety in low-income communities, and identification of influential family processes on firearm outcomes is a major gap in quantitative literature [57, 58]. Socialization is the process by which individuals learn norms and behaviors that allow them to engage successfully in their community [59]. Qualitative studies could provide insights into the ways families teach their children to stay safe in neighborhoods where there are high rates of violence, as well as youths’ own beliefs about how to engage in the community in a way that keeps them safe. Learning which messages, beliefs and subsequent behaviors are effective for avoiding or abstaining from violence may be important in developing social messaging campaigns to shift community norms about violence or created targeted prevention programming.

Finally, it is important to keep in mind that the experiences with community firearm violence as reported by community members of low-income communities are truly unique compared to middle- or high-income communities. Firearm violence in these contexts is the direct result of systematic disinvestment and societal racism (e.g., resource deprovision) and as a result, alleviating these processes of marginalization is the most necessary intervention. Prevention of violence occurs systematically, not individually, and seeking the voices and perspectives of individuals in these communities is critical for their reinvestment (Author reference).

#### Strengths of the Review

This scoping review had several strengths. First, this review compliments and extends existing quantitative reviews of firearm violence exposure and behavior by summarizing relevant qualitative findings. Prior reviews focused on firearm violence and related factors have been published, but a summary of current qualitative methods and findings on the attitudes, behaviors, causes, and consequences of firearm violence in low-income communities had not yet been conducted. Second, the review did not isolate the inclusion criteria to any one developmental period or geographic setting. Instead, this study sought to identify studies of experiences with and perspectives on community firearm violence exposure in low-income communities as a way to include all demographic features of communities that are most impacted by firearm violence. Third, this review utilized a developmentally considerate model for prevention of violence, the Social-Ecological Model, as a framework for summarization and categorization of current qualitative community firearm violence research. Finally, this review supported the findings of prior quantitative reviews of firearm violence exposure and carriage, sharing agreement that risk factors, particularly those at the individual level (i.e., gun carriage, possession, motivation for violence), have been the focus of studies on these topics to date [57, 58, 60]. Interestingly, the qualitative results discussed in this review provided further insight into community and societal factors (i.e., experience as a gunshot wound patient, perspectives of police), they also shared specific community-member reported recommendations for managing and attenuating gun violence in their communities. The richness, nuance, and opportunity for learning about community perspectives provided qualitative research is invaluable and will be necessary to move the field, and society, forward. That said, a focus on broader societal influences, social media, and pathways or mechanisms from risk and promotive factors to firearm related outcomes are absent from this study as well as prior reviews. This review corroborates that an ongoing focus on inherent strengths, promotive, and protective factors will be important for moving this body of literature forward and improving the quality and development of intervention and prevention practices.

#### Limitations of the Review

This review has several limitations related to the scoping review design and generalizability of findings. Though it is a strength of this scoping review to use strict inclusion and exclusion criteria, the studies that were reviewed were limited to those that reported their sample or setting as low-income. During the in-text review, a significant number of studies were ultimately excluded due to lack of information about the socioeconomic status of their study setting or participants, in some cases because the authors or researchers elected to not collect demographic information to protect the identities of their participants. In several cases (*n* = 8) studies appeared to focus on low-income communities without explicit statement. In these cases, the lead author reached out to each studies’ corresponding author to request confirmation or denial of sample demographics. In all cases but one, the corresponding author responded, which allowed the lead author to include or exclude the article. Second, it is likely that not all qualitative methodologies were captured by the search terms. For instance, photovoice, text and document analyses were not included in the search terms and thus it is not a surprise that these methods were not represented in the reviewed studies. Finally, any of the reviewed studies stand alone in their sampling and findings, for instance the article by Hitchens [45] was the only article reviewed that focused on the experiences of firearm-homicide loss by street identified Black women and girls. As such, findings from this review should be used as a guide rather than be considered a quintessential synopsis of experiences for low-income communities.

#### Limitations of the Literature

The reviewed literature poses several limitations. The first limitation is regarding sampling. The majority of studies included in this review used adult samples and while there were a few studies that included youth samples, and young adults were included in both youth and adult samples. Young adulthood is a unique period of increased responsibility both personally (i.e., role changes, financial independence) and legally (i.e., viewed as an adult by society, though developmentally not far off from adolescence). Thus, it may be important to specifically focus on the experiences of young adults in these contexts. Second, homogenous racial groups outside of Black and African American communities were not considered by studies in this review, despite empirical knowledge that other minoritized racial and ethnic groups experience high rates of firearm violence. Third, most studies included all, or mostly male samples, limiting what is known about the experiences of female gun violence victims, relatives, and peers. Finally, few studies considered the influence of peer opinions on an individual's behavior, and no studies considered the influence of familial, societal, or political messages about guns or violence on individual behavior and opinion of guns. The studies included in this review span many firearm related topics across the levels of the Social-Ecological Model, and the limitations reported are an opportunity for future research.

#### Future Directions

At the individual level, it may be helpful to understand changes in behaviors, motivations, beliefs, and perceptions across developmental periods (e.g., early adolescence to late adolescence, adolescence to young adulthood). One focus for future research may be to further explore the experiences and elements of survivorship for those who have been directly (e.g., been shot) or indirectly (e.g., family member or friend of a person who has been injured or killed) exposed to firearm violence. Additionally, qualitative methods may be used to further explore the intricate dynamics of peer, family, community and societal factors that influence individuals’ and communities’ experiences with firearm violence. Specifically, it may be helpful to understand peer and family (e.g., caregivers, sibling, family-figures) influence on perceptions and engagement in community firearm violence for specific developmental periods (e.g., older adolescents and young adults), as well as to consider these groups’ influence on *lack of* engagement in community firearm violence or exposure to firearm violence. Additionally, because low-income communities are plagued by firearm violence because of systemic disinvestment and historic and current racism, it is important for future research to focus on developing a deeper understanding of the ways in which community members wish to see these societal factors be thwarted.

## Conclusion

Firearm injury and death disproportionately impacts people in low-income communities, in particular Black and male youth and adults [2]. The Social-Ecological Model was used as a framework for prevention and guided the findings of this study. Understanding the existing literature, categorized by levels of the model (e.g., individual, relationship, community, society), is important for identifying targets for intervention and prevention efforts. In addition to summarizing the emergent literature, this review offers considerations for future community firearm violence research to broaden the focus to a wider range of highly-impacted populations with in low-income settings, begin to specify antecedent for firearm behavior and outcomes, consider processes of socialization with firearms and safety, and incorporate community knowledge in the development or modification of social and built environment interventions. Qualitative methods are an important piece to the community firearm violence prevention puzzle and should be utilized as an empirical strategy to involve the perspectives and experiences of low-income communities who are deeply impacted by firearm violence.

## Data Availability

The studies reviewed, and data derived during the review may be made available to the public.
